# Indents

**Published:** 1921-09

**Authors:** 


### INDENTS

**☞Brief Articles of Technical or Scientific Value will receive notice and comment. Contributions invited.**

---

Children in Practice. In treating children of nervous parents I have found it expedient to attempt the re-education of the parent. That seems to be the best and quickest way to reach the child because there seems to be a strong nervous reflex between the parent and child. Until the parent has been made to understand, the operator will find great difficulty in controlling this type of child. One speaker referred to the matter of bribing children into bravery. It has never been my practice to tempt a child to do anything for a bribe. The fact is I would not take a bribe myself and I feel that the child should be taught to stand upon his own feet, to cultivate a courage of his own, and to learn to do things because they should be done and because it is right that they should. A reward may perhaps be looked at in a different sense. A reward comes without expectation because one has done a certain thing in the right way or has acted properly. A bribe is something that is offered to a person to do something that he would otherwise not do. You can understand why I eliminate entirely this sort of thing from my practice. If you can obtain the right mental attitude and teach the child to see the things in the right light, bribing is never necessary.

I mentioned the attributes of love, truth, kindness, confidence, and trust. One may train himself to habitually act in accordance with the principles of kindliness. It then becomes quite natural to treat an unlovable child in such a way as to gain his confidence and trust. Thus the whole matter works out better than might otherwise be the case. I think psychology should be placed on the curriculum of the dental schools. The trouble is in finding a psychologist who understands our problems. It will be best for us to study, observe, and finally evolve our own psychological literature. In this way I believe we shall be more apt to establish ourselves upon the right basis. It was interesting to hear from Dr. Motley who, having had training as a teacher, is in a position to speak with authority upon this subject. What he said encouraged me greatly. Some of us go through life engaged in daily practice scarcely giving thought to the importance of the subject of psychology. Undoubtedly the more skilled one becomes in the use of psychological principles the more useful he will be.—Dr. A. P. Rogers, in *Journal, N. D. A.*, Aug., 1921.

---

THE ETHYL-CHLORID SPRAY FOR INFLAMMATION AND PAIN.

To reduce inflammation and stop pain, the ethyl-chlorid spray has been found invaluable. Where the tissues have not been broken or lacerated the parts are sprayed until frost just begins to form, and this repeated several times. When the tissues have been lacerated the spray should be applied as near as possible without getting it on the raw surfaces as this would cause great pain for the moment. Sensitive teeth should be protected by wax or modeling compound. Frequently the spray is applied externally. The effect is quite permanent (that is, not merely anesthetic) and the relief from pain often almost instantaneous.—Frederick W. Stephan, Chicago.

TAKING A BITE FOR CROWN OR BRIDGE WORK. One of the most difficult items in operative dentistry to contend with is the taking of a satisfactory bite, where the occlusion is extremely close. The following is submitted for its possible assistance to the operator in such cases. When the wax or compound bite is removed it often breaks in two pieces, making it difficult to get a perfect cast for the occluding surface or surfaces to be articulated to the crown or bridge to be constructed; or breaks when placed on the plaster model. A good, strong, sharp bite can be obtained in the following manner: Take a piece of linen which comes on each side of a sheet of vulcanite rubber, cut it about length and width of bite to be taken, place bite wax above and below the piece of linen, and take bite in usual manner. The linen holds the bite together and prevents patient from biting clear through. Do not use paper, as paper becomes wet and tears.—H. F. Schlieffarth, *Dental Facts.*

---

SCRUTINIZE COLLECTION AGENCY CONTRACTS. Dentists should be somewhat cautious of collecting agencies if they desire to avoid unpleasant complications. Perhaps the average dentist places nothing but ''deadbeat'' accounts with collecting agencies, and really does not care what happens, or for that matter whether the account is ever collected or not. The possibilities of getting into serious embarrassment should not be overlooked, for not infrequently the collecting agencies take unfair advantage of their position and place the dentist in an embarrassing position through misrepresentation. Not infrequently the dentist is made the victim of an unfair advantage through some such joker as follows:

''In the event the undersigned should withdraw any claims after having assigned the same to said association, the commission thereon shall be paid to said association the same as if said association had collected such claims in full.''

It is all right to protect the collecting agencies from withdrawal of accounts immediately after pressure has been brought to bear upon the debtor which results in payment direct to the dentist, but the dentist is not protected if after a suitable length of time there are no results obtained by the collecting agencies and it is desired to place the accounts in other hands for collection. There is no occasion for making a contract that is one-sided in its protection.—Editorial in *Journal, N. D. A.*

---

MAXILLARY X-RAY LANDMARKS. A great deal has been written in regard to the making of X-ray exposures of the teeth and every radiographer is familiar with the idea of bisecting the angle between the plane of the film and the plane through the long axis of the upper teeth. In carrying out this idea the technic for the lower arch is very simple, as there can be no doubt about the general direction of the teeth. However, in the upper arch the question is more difficult and it takes a student of comparative anatomy to determine the direction of the various teeth and even with such students time is consumed in establishing the angles.

To facilitate the X-ray operation and to overcome the necessity of studying the relation of the teeth and the film in the mouth, the lower half of the ridge of the nose can be used as an anatomical landmark, providing it has not been disturbed in previous times by the impact of a baseball bat or the like. For all dental X-ray purposes that portion of the nose from the ''hump'' down to the end or, in other words, the ridge of the cartilage, is parallel with the long axis of the central incisors and a plane drawn through this line of the ridge can be placed parallel with a similar plane passed through the long axis of any of the upper teeth. Now, in using this ridge and an imaginary plane to it as an external landmark is a much easier process than it is to look into the mouth and guess at the direction of the teeth. In order to get a general direction of the film, place a pencil or tongue applicator back of the centrals and the angle where the pencil intersects the projected line of the nasal ridge will be the angle of bisection that we are trying to obtain. Now by placing the edge of the compression cone of the X-ray machine against the junction of the eyebrows so placed that a cross section of the cone is at an angle with a plane through the nasal ridge equal to one-half the angle to be bisected. The rays will now throw a shadow on the film an exact length of the upper centrals. To obtain the rest of the upper arch, all that is necessary to do is to rotate the head or the machine (depending upon the make) with the edge of the cone against the eyebrow and around to the temple, at the same time keeping the same angle of the cone to the nasal plane while the nasal plane has been rotated until it is parallel with the long axis of teeth desired to be radiographed. This may sound complicated to a party not interested in plane geometry but there is no more concise or simpler way to explain it, and I am sure it is far more exact and quicker than looking into the mouth and there is no machine so calibrated that it can possibly obtain the correct angles.

In the fewest possible words, let me put it in another way. First, let the X-rays fall perpendicular upon a plane passing through the ridge of the nose and which plane is parallel with a similar plane through the long axis of the tooth to be radiographed, and then rotate the head (chin toward the neck) equivalent to one-half the angle of bisection between the film and tooth, always keeping the eyebrow or temple on the edge of a flat end compression cone.

There has been considerable discussion recently in regard to the zygomatic arch and the Malar process, the general trend seeming to claim that the usual shadow thrown over the roots of the second upper molar is of the heavy bone. As a matter of fact we are not nearly as concerned about the zygoma as we are about the thickened floor of the antrum and it is a far greater problem to eliminate the obliteration of the roots of the upper second molar, due to the floor, than it is to dodge the facial bone, and very often an error is made in claiming that the obliteration is due to the shadow of the zygoma when really it is the thickened floor of the antrum. It should be remembered that the anatomy of the antrum as far as the X-ray is concerned is shaped much the same as a deep saucer with the open side facing the upper bridge of the nose and if the upper second molars are taken in the usual way the rays have to penetrate not only the bottom of the saucer but from edge to edge and through both sides as well. Now, in order to avoid this condition it is necessary to penetrate the floor of the antrum at an angle of about 20 or 30 degrees by directing the rays from the forward direction. When this direction has been observed and carried out with the necessary angles as indicated by the nose, usually the three molars and two bicuspids will be shown on the same film. · However, it must be remembered that as a rule a film is not of much value for more than two or three teeth and that 12 to 16 exposures should be required to be of complete diagnostic value.—Dr. W. H. Thwaits, in *Dental Facts.*

---

Taking Impression of a Cavity. To get impression for porcelain inlay, where cavity extends under the gum line, prepare cavity as usual. Bend piece of rigid metal to make a half cylinder, then cut end of said half cylinder to fit convexity of the tooth neck. Have modeling composition ready, warmed, on a stiff spatula-shaped instrument, push gum back with this metal until upper edge of the cavity is exposed, reheat modeling composition and press into the cavity. The metal forms a matrix which keeps the modeling composition from spreading around under pressure, and gives a very fine, clean-cut impression.—C. E. Allshouse, *Dental Facts.*

